# In-Situ Crystallization and Characteristics of Alkali-Activated Materials-Supported Analcime-C from a By-Product of the Lithium Carbonate Industry

**DOI:** 10.3390/ma15031261

**Published:** 2022-02-08

**Authors:** Lixiang Huang, Le Han, Ze Liu, Jixiang Wang, Yanbo Zhang, Dongmin Wang

**Affiliations:** 1 School of Chemical and Environmental Engineering, China University of Mining & Technology, Beijing 100083, China; hlx2575@163.com (L.H.); hanle19960423@gmail.com (L.H.); wjxiang121@gmail.com (J.W.); wrzhangyanbo@163.com (Y.Z.); wangdongmin@cumtb.edu.cn (D.W.); 2 Xi’an Research Institute of China Coal Technology and Engineering Group Corp, Xi’an 710054, China

**Keywords:** alkali-activated materials, analcime-c, crystallization, by-product of lithium carbonate industry

## Abstract

The present study proposes a new process for synthesis of alkali-activated materials (AAM)-supported analcime-C foam materials (AFs), utilizing a by-product of the lithium carbonate industry. This material has great application value as a bulk-type solid adsorbent. Characterization analyses show that the alkaline activator modulus greatly affects the crystallinity of analcime-C in AFs. Furthermore, the compressive strength, zeolite yield, and microstructure of AFs are significantly affected by the saturated steam parameters, including crystallization pressure, temperature, and time. The synthesized materials comprise pores of different sizes (micro to macro). They combine the functional micro-porosity of the analcime-C, the meso-porosity of the gel matrix, and the macro-porosity of the foamed AAM. The maximum compressive strength, density, total porosity, and Pb^2+^ adsorption capacity of AFs investigated in this study are 1.15 MPa, 350 kg/m^3^, 76.5%, and 69.3 mg/g Pb^2+^, respectively. Unlike many granular adsorbents, the bulk AFs adsorbent produced by this process is easy to recycle. In addition, it also contributes to the comprehensive utilization of a by-product of the lithium carbonate industry.

## 1. Introduction

In recent years, the demand for lithium products has appreciably grown. In particular, lithium ion batteries have become very popular due to their use in many applications related to the conservation, storage, and transmission of renewable energy [1]. The main method currently employed in the extraction of naturally occurring lithium at an industrial level uses the calcium carbonate method. A major disadvantage of this method is the formation of a solid waste lithium slag (LS), characterized by high water absorption capacity, low activity, and large SO_4_^2−^ content [2,3]. Such unfavorable characteristics render the utilization of LS difficult, which ultimately results in its accumulation, leading to environmental pollution, land occupation, and waste of resources. Therefore, it is necessary to either utilize or dispose of the increasing amounts of LS solid waste. At present, LS is mainly used as a concrete admixture in building materials due to its filling effect and agglomeration effects [4,5,6]. Studies have shown that when added in the right amount, LS may improve the compressive strength, drying shrinkage, modulus of elasticity, and creep properties of concrete [5]. Furthermore, mechanical grinding of LS increases its specific surface area, thereby promoting the hydration of concrete to produce ettringite, calcium sulphate, and calcium silicate hydrate (C-S-H) gel [4]. Unfortunately, the high sulfur content in LS limits its use in the design of traditional concrete [7].

Alkali-activated materials (AAM) are developed from a mixture of an amorphous aluminosilicate precursor and an activating solution at ambient or slightly elevated temperature (T < 100 °C) [8,9]. It generally exhibits excellent thermal stability, high strength, low shrinkage rate, outstanding fire resistance and long service life [10,11]. LS, furthermore, mainly consists of amorphous aluminosilicate, crystalline aluminosilicate, gypsum and quartz, and thus, it has a certain pozzolanic activity [5]. The great advancement achieved recently in the field of AAM [12] has made it possible to prepare LS-based AAM. To increase the amorphous content of these materials, LS is usually calcined and modified at 700 °C prior to alkali activation [13].

Foam alkali-activated materials (FAAs) is an inorganic foam material with high porosity, low density, and well mechanical properties [14,15,16]. The most common synthesis route to produce FAAs is by the incorporation of a foaming agent (e.g., hydrogen peroxide, fine metallic powders) [17,18] into the AAM slurry, usually known as the chemical foaming technique. This strategy takes advantage of in-situ reactions of the foaming agent in the alkaline medium, inherent in the alkali activation of aluminosilicate precursors. This process generates gas bubbles which are then trapped inside the slurry during setting, leading to the production of voids in the hardened body [19,20]. FAAs are often used to manufacture external wall insulation boards with their excellent thermal stability and low thermal conductivity [21,22]. Circulating fluidized bed fly ash and hollow microbeads are used as raw materials to produce high-performance foaming materials with low thermal conductivity (0.05 W/(m^2^/K)), low density (200 kg/m^3^) and high strength (compressive strength 1.06 MPa) after alkali activation and chemical foaming [14].

In recent years, research on synthetic zeolites has become more and more extensive. Zeolite is an inorganic nano-crystalline mineral with a tetrahedral aluminosilicate skeleton, which possesses high specific surface area and porous structure, making it a kind of efficient adsorbent [23]. There are many reports [24,25,26] on the use of industrial solid waste (fly ash, LS, etc.) to synthesize zeolites. Currently, the main synthesis method is the hydrothermal method, which has advantages of low energy consumption, easy control, and pure phase [23]. The zeolites synthesized from industrial solid waste include Na-P, analcime, and faujasite [27]. They are used as soil improvers, heavy metal ion adsorbents, and volatile organic compounds (VOC) adsorbents which greatly increase the added value of industrial solid waste and contribute to environmental protection [27].

AAMs are generally considered to be the precursors to zeolites [25]. In the preparation of AAMs, small amounts of zeolite phase are often detected. As the temperature and pressure increase, the zeolite phase gradually increases [27]. It has been reported that mesoporous AAMs have been prepared by the hydrothermal method [26,28,29], the mesoporous AAMs not only exhibited a certain strength, but also supported Na-P zeolite, faujasite, sodalite, etc. Higher porosity and surface area were achieved for the AAM-supported zeolites. These materials can potentially be used as membranes/filters for removal of hazardous contaminants from water owing to their porous structure and the affinity of zeolites towards cations [25].Hammad R. Khalid et al. [28] used slag and fly ash as raw materials to synthesize zeolite-loaded AAM by the hydrothermal method. Meanwhile, they used the prepared material to adsorb heavy metal ions Pb^2+^. The adsorption results shown that zeolite-loaded AAM had a high affinity for Pb^2+^, showing 37.9 mg/g. Its adsorption capacity was much higher than traditional geopolymer materials (6.3 mg/g) [29,30].

This paper proposes a saturated steam treatment method to synthesis of AFs. The study mainly discusses the effect of different modules of sodium water glass and saturated steam treatment time, pressure, and temperature on the mechanical strength, analcime-C crystallinity, and pore structure of the AFs. At the same time, the AFs were subjected to N_2_ adsorption and mercury intrusion experiments (MIP) to characterize its pore structure and evaluate its adsorption capacity. Finally, the AFs were used for Pb^2+^ adsorption experiments to evaluate their adsorption capacity for Pb^2+^.

## 2. Materials and Experiments

### 2.1. Resource Materials

**Figure 1:**
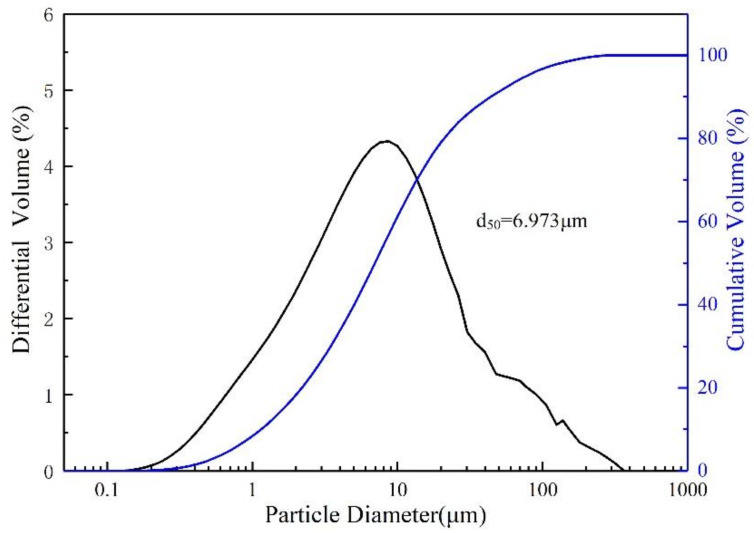
Particle size diameter distribution of MLS.

LS from Tian qi Lithium corporation (Sichuan, China) was discharged after the sulfation method. LS has low reactivity and requires mechanical and chemical activation. The as-received LS was ground for 30 min, and then calcined in a muffle furnace at 700 °C for 2 h, with a heating rate of 5 °C/min. After being cooled to room temperature, modified lithium slag (MLS) was obtained [13]. The chemical composition of the MLS, analyzed by X-ray Fluorescence (XRF) is shown in Table 1. The distribution of particle size diameter is shown in Figure 1.

The modulus of water glass (from Hongxing, China) was around 2.42 (SiO_2_/Na_2_O molar ratio = 2.42). The role of adding water glass was to provide sufficient Si and improve the formation of AAM precursors [31]. Sodium hydroxide of analytical reagent (AR) purity was used to adjust the modulus of water glass. H_2_O_2_ solution (35 wt%) and calcium stearate powder were introduced as a chemical foaming agent and stabilizing agent, respectively. Studies [14,19] have shown that these two reagents are an excellent foaming agent and foam stabilizer.

### 2.2. Experimental Procedures

**Figure 2:**
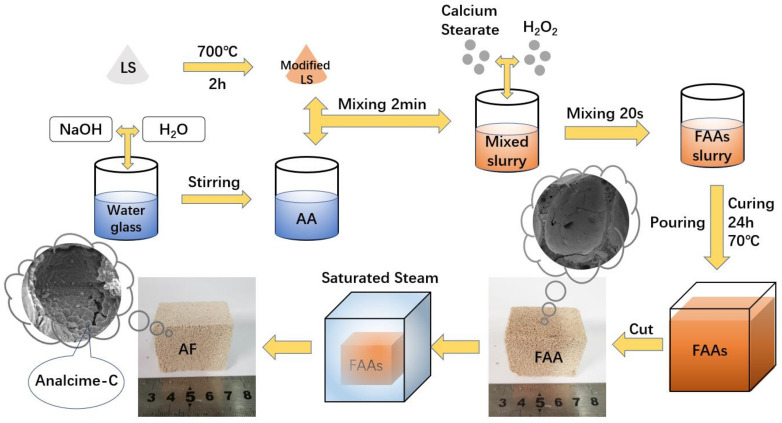
Schematic illustration of preparation procedure for AFs.

A flow diagram for the preparation of AFs is presented in Figure 2. Based on previous research [32], firstly, sodium hydroxide, water glass, and H_2_O were mixed to obtain the required modulus and concentration of the AA, as given in Table 2. Secondly, 400 g MLS powder was weighed, mixed with AA, and stirred for 2 min. After that, H_2_O_2_ and calcium stearate were added into the mixed slurry (The calcium stearate and H_2_O_2_ occupied 0.25 wt% and 2.5 wt% of the MLS, respectively). Then, the obtained FAAs slurry was cast into 40 mm × 40 mm × 160 mm molds and coated with polyethylene thin films to prevent gain or loss of moisture. Then, the molds were cured at 70 °C. After 24 h, the cured FAAs synthesized under different modulus conditions were demolded and transferred to a saturated steam chamber with different saturated steam conditions. All samples were dried at 60 °C for characterization.

### 2.3. Characterization

The compressive strength of the samples was measured using a CS200 universal servo stress tester (maximum load 500 KN, Ningbo Weiheng, China), the loading speed was maintained at 12 KN/min, and the contact area was 40 mm × 40 mm. X-ray diffractometer (XRD) analysis was carried out on a Shimadzu X-ray diffractometer (XRD-7000, Shanghai, China) with Cu-Kα (1.5406 Å) radiation at 40 kV and 40 mA. Powder samples were subjected to Cu Kα radiation at a scan speed of 10°/min from 10° to 60° (2θ). Scanning electron microscope (SEM) analysis was performed using a ZEISS SIGMA 500 (Shanghai, China) (Acceleration voltage: 0.2~30 kV). Selected samples were cut into 5 mm × 5 mm × 0.5 mm sheet specimens with a relatively flat surface. The porous structure of samples was characterized by automatic high-performance mercury intrusion meter (Micromeritics apparatus (Autopore III, America)) and automatic surface area device (Micromeritics apparatus (ASAP2460, America)), samples were cut into 3 mm × 3 mm × 3 mm rectangles. The parameters of the macro-porosity were measured by mercury intrusion porosimeter (Mercury parameter: surface tension 485 dyn/cm, contact angle 130 degrees, mercury density 13.5335 g/cm^3^). The parameters of micro-porosity and the meso-porosity were measured by analyzing the N_2_ adsorption/desorption isotherms at −195.85 °C. The density functional theory (DFT) method was used to calculate the pore volumes and pore size distribution. Total porosity of the foams was obtained from the bulk density and the theoretical density ratio with Equation (1)

(1) $$\mathrm{Tp}=\left(1-\rho_{r}/\rho_{0}\right)\times 100\%$$

where Tp represents the total porosity of the foams, $\rho_{r}$(=1500 kg/m^3^) and $\rho_{0}$ are the dry bulk density and dry density of the FAA samples, respectively [19].

### 2.4. Rietveld Quantitative Analysis

We added 20% standard TiO_2_ as an internal standard material, and the amount of corundum was accurately recorded for each measurement. The mixed samples to be analyzed were carefully ground in an agate mortar with ethanol added to promote particle dispersion. The mixture was then dried at 60 °C for characterization.

Rietveld [33] applies GSAS software with a pseudo Voigt function to refine powder patterns. The crystal structures used to interpret the powder patterns were taken from the Inorganic Crystal Structure Database (ICSD). The collection codes for each structure are: analcime-C # 15881, quartz # 42498, spodumene # 69394/lithium aluminum silicate # 14235, calcium sulfate # 24473, sodium sulfate # 31687, rutile # 9161. The parameters optimized were thermal vibration parameters, background coefficients, cell parameters, zero-shift error, peak shape parameters (including anisotropic terms if needed), and phase fractions.

### 2.5. Adsorption Tests

The adsorption efficiency of AFs was evaluated by Pb^2+^ adsorption experiments. Samples were cut into 20 mm× 20 mm × 20 mm rectangles using a precision cutter and the exact quantity of AF was recorded for each measurement. According to the research of analcime-C adsorption [34,35], 300 mL starting solution of 1000 mg/L Pb^2+^ concentration was used to explore the full potential of AFs. The pH of the starting solution was set at 4. After contact with the AFs, test solution was shaken for specified times (1, 3, 6,12, and 24 h) at 25 °C and 200 rpm, then the suspensions were centrifuged. The supernatant was filtered through a 0.2 μm syringe filter, and residual concentration of Pb^2+^ was measured using ICP-OES (PerkinElmer 8300, Shanghai, China). The pseudo-first-order kinetic and the pseudo-second-order kinetic models were employed in this study. The adsorption amount by the adsorbent was calculated using Equation (2)

(2) $$q_{t}=\frac{\left(C_{0}-C_{t}\right)\times V}{M}$$

where q_t_ is the adsorption amount by the AFs adsorbent, C_0_ and C_t_ are the initial and measured concentrations of the heavy metal in the liquid phase at time t, M is the mass of AFs, and V is the volume of the heavy metal solution [34].

## 3. Results and Discussion

### 3.1. The Effects of Synthesis Parameters on Crystallization of AFs

**Figure 3:**
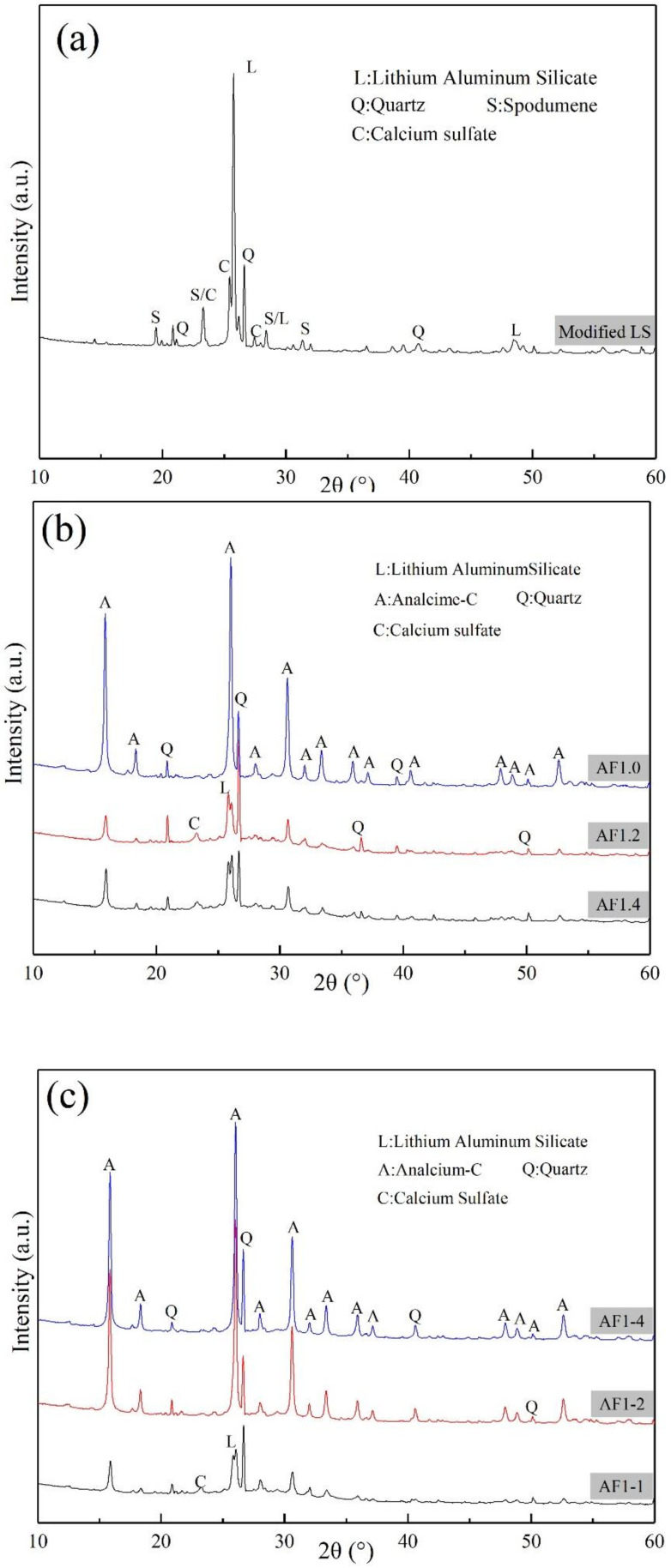

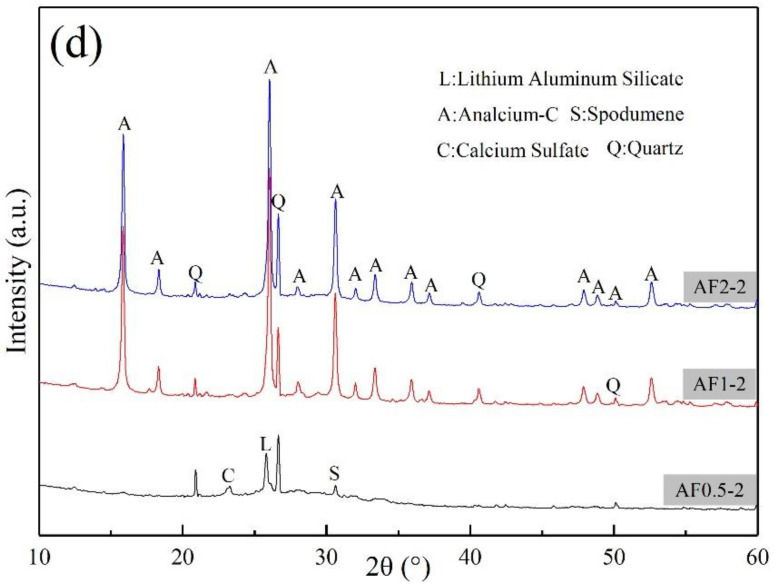
XRD patterns: (**a**) MLS, (**b**) AFs with different AA modulus, (**c**) time, (**d**) pressure.

As shown in Figure 3a, peaks corresponding to lithium aluminum silicate (PDF#21-0503), quartz (PDF#85-0795), spodumene (PDF#71-1063), calcium sulfate (PDF#70-0909) were identified in MLS, similarly to those observed in previous studies [36,37].

The newly formed crystalline phase in AFs (Figure 3b–d) was analcime-C (PDF#76-0902). Since Na-based AA was used in this study, the formation of analcime-C was evident [38,39,40]. The lower AA modulus, longer crystallization time, and higher temperature make the crystalline phases except quartz gradually decrease until they disappear, greatly improving the crystallization of the analcime-C. Although the AAM gels cured at high temperature tend to develop rapidly [41,42], under saturated steam treatment, a large amount of N(C)-A-S-H of FAAs was converted to analcime-C, resulting in a decrease in the amorphous phase. Compared with many studies on self-supporting zeolite materials [30,31,43], the saturated steam treatment method was found to be similar to the general hydrothermal method. The traditional hydrothermal method required the samples to be in contact with water, and the tumbling boiling water could destroy the fragile FAAs during the crystallization process, resulting in disintegration of FAAs. However, saturated steam treatment method could maximally retain the macro structure of the samples, and increase the yield of the analcime-C.

**Figure A1:**
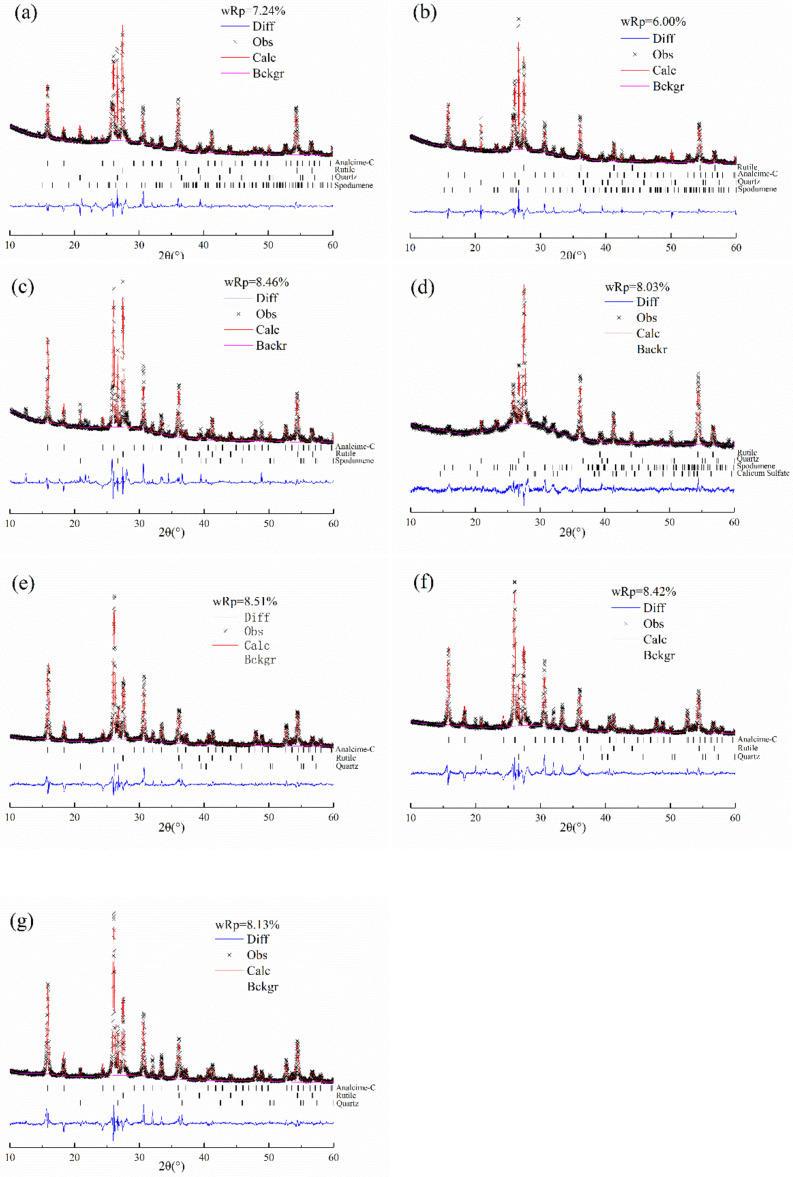
Rietveld quantitative analysis of (**a**) AF1.4, (**b**) AF1.2, (**c**) AF1.0 (AF1-2), (**d**) AF0.5-2, (**e**) AF2-2, (**f**) AF1-1, and (**g**) AF1-4.

Rietveld quantitative analysis demonstration is shown in Figure A1, and Table 3 shows the Rietveld quantitative analysis results of different condition AFs. The yield of analcime-C varies significantly in samples AFn1.4, AFn1.2, and AFn1.0 under the saturated steam conditions, which might be due to the difference in structural units of AAM gels. As the pressure increased from 0.5 MPa to 2 MPa (151.85 °C to 212.37 °C, respectively), the content of analcime-C increased from 0% to 48.89%. At the same time, with the crystallization time increasing, the content of analcime-C increased from 13.94% to 45.21%, showing that the temperature and pressure were crucial in the crystallization of analcime-C. Moreover, it was interesting to note that the content of lithium aluminum silicate and sulfate compounds, which are normally considered hard to completely react in FAAs, significantly reduced in samples AFn1.0, AF1-4, and AF2-2. It suggested that the high temperature and pressure could change these lower reactive phases to other products.

### 3.2. The Relationship between Compressive Strength and Analcime-C Yield of AFs

**Figure 4:**
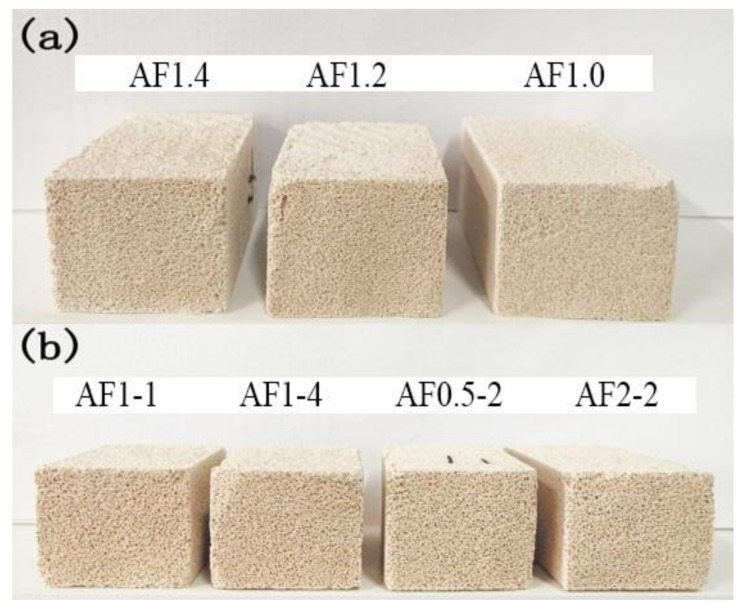
Optical photographs of AFs with (**a**) different AA modules and (**b**) different pressure and time.

**Figure 5:**
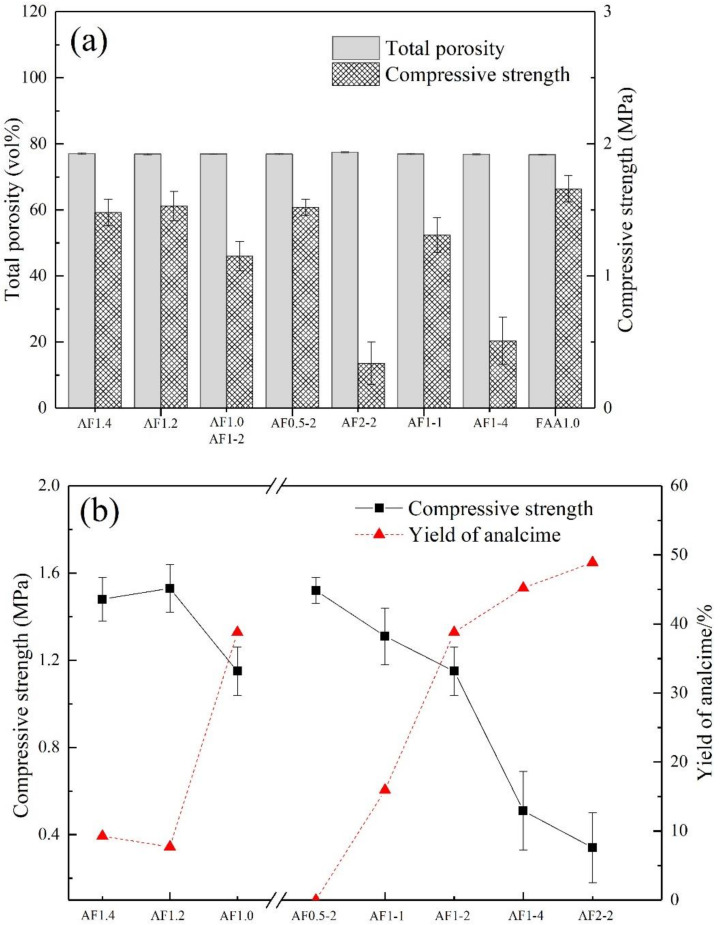
(**a**) Total porosity, compressive strength, and (**b**) zeolite yield results of AFs.

In order to compare the effect of saturated steam conditions on FAAs more intuitively, we introduced a blank control group FAAs1.0. As shown in Figure 4 and Figure 5a, the AFs were approximately khaki color, and they showed similar total porosity (approximately 76.5%). In contrast, different synthesis conditions have a greater impact on the compressive strength. As the AA modulus decreases, the compressive strength of AFs shows a downward trend, and the compressive strength also decreases significantly with increases in pressure and crystallization time. And the compressive strength of the samples AF2-2 and AF1-4 were only 0.34 MPa and 0.51 MPa, decreases of 79.14% and 68.71%, respectively, compared to the samples without saturated steam treatment (FAA1.0). This could be attributed to the crystallization process causing damage to the pore structure, which will be discussed in the porosity structure analysis. At the same time, we found that when the compressive strength was lower than 0.5 MPa, the sample was unstable, and it was easily deformed during drying or cutting, which might prove troublesome in the application of AFs.

Therefore, we must comprehensively consider the production of analcime-C and its compressive strength to obtain AFs with the best performance. As shown in Figure 5b, with the pressure and crystallization time increased, the yield of analcime-C gradually grew, while the compressive strength decreased greatly. Obviously, AFn1.0 (AF1-2) showed better performance, both in compressive strength and the yield of analcime-C.

### 3.3. The porosity Structure Analysis

**Figure 6:**
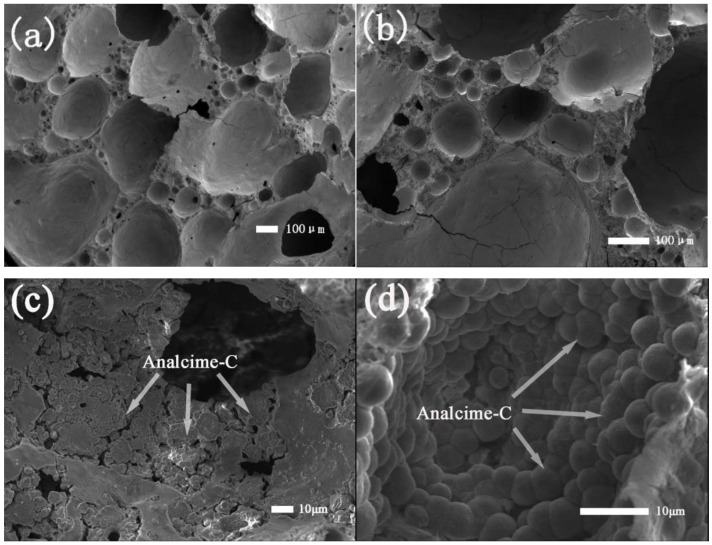
SEM images of (**a**,**b**) FAA1.0, and (**c**,**d**) AF1-2.

In the SEM image, it can be clearly observed that the pore walls of the blank sample FAA1.0 (Figure 6a,b) are relatively smooth and dense, most of which are large pores with a pore diameter of more than 300 μm, which determines the porosity of the material, and provides a good growth environment for the in-situ loading of zeolite. After saturated steam treatment, they maintain the original spatial structure. As shown in Figure 6c,d, the growth of analcime-C destroys the original pore walls, resulting in a large number of cracks and pores, which greatly enriches the porous structure of the material.

**Figure 7:**
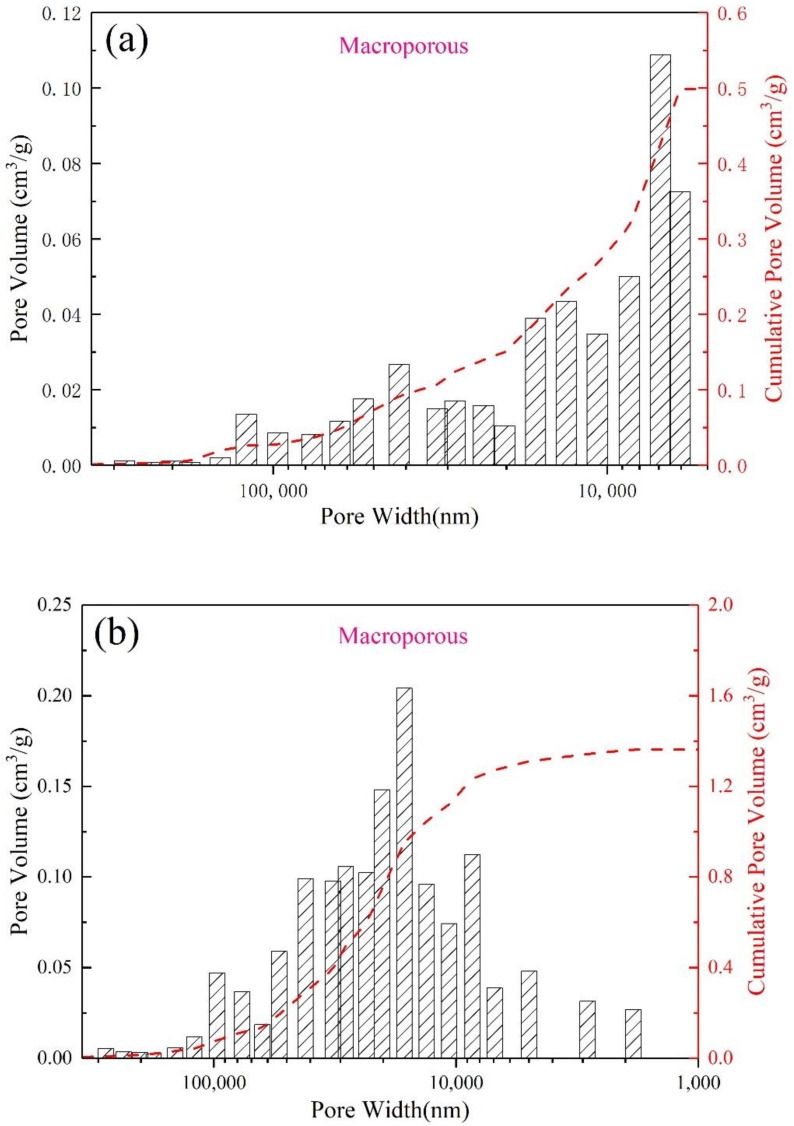

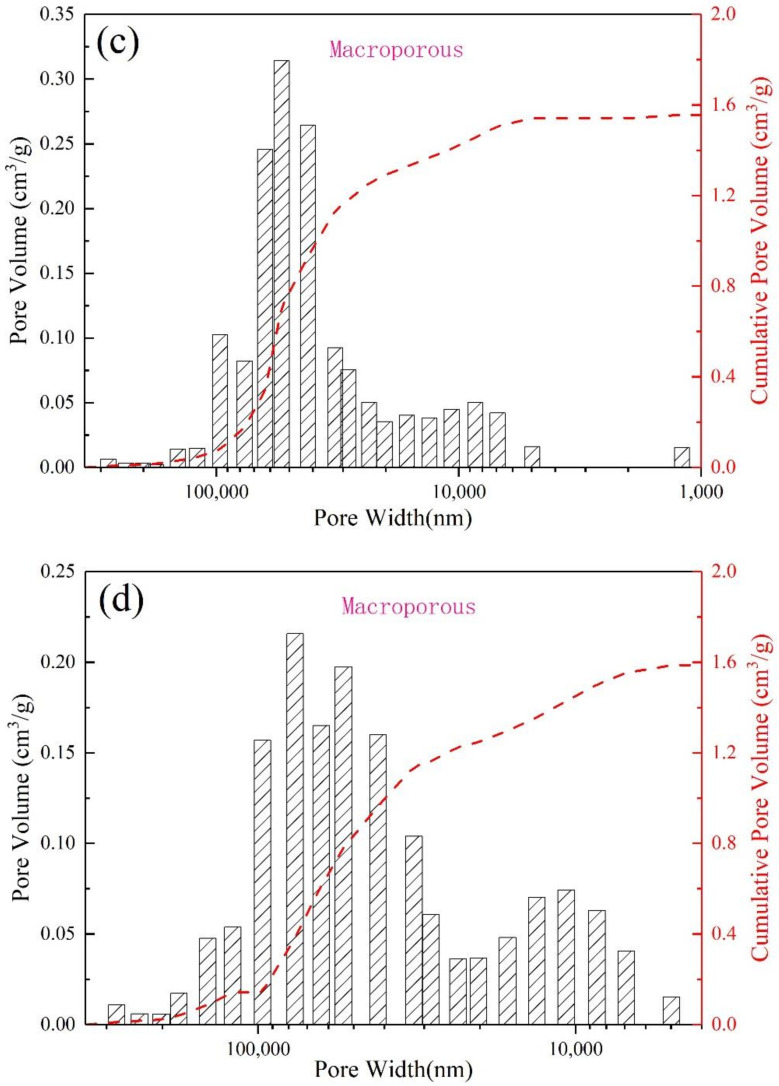
MIP characterization for (**a**) FAA1.0, (**b**) AF0.5-2, (**c**) AF1-1, and (**d**) AF1-2.

**Figure 8:**
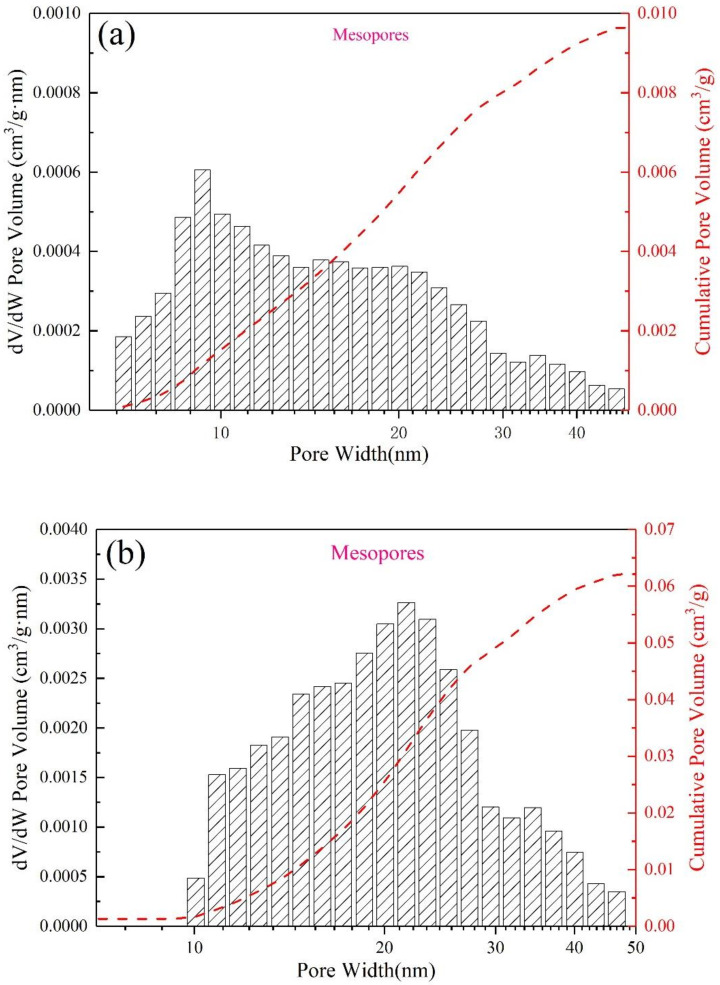

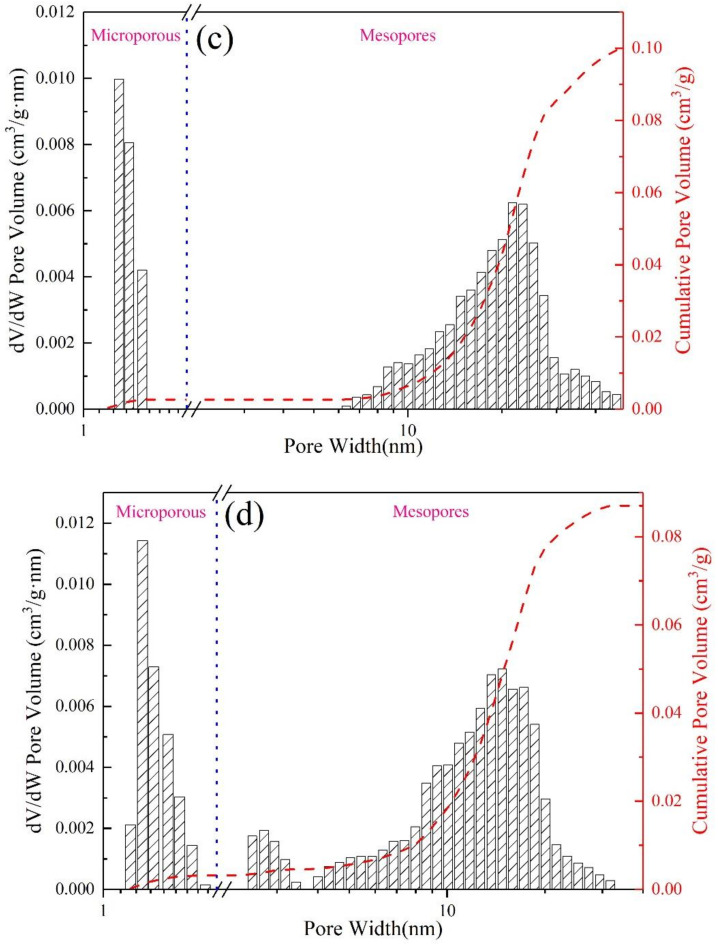
DFT pore size distribution characterization for (**a**) FAA1.0, (**b**) AF0.5-2, (**c**) AF1-1, and (**d**) AF1-2.

**Figure 9:**
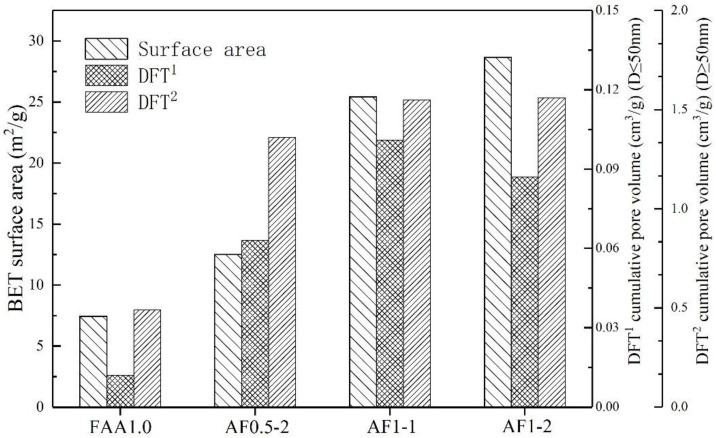
BET surface area and DFT cumulative pore volume characterization for FAA1.0, AF0.5-2, AF1-1, and AF1-2.

In order to more likely show the effect of different saturated steam conditions on the pore structure of AFs, in the following analysis, we selected four groups of FAA1.0, AF0.5-2, AF1-1 and AF1-2 samples with more obvious yields of analcime-C. The large pore size distributions of the four samples were obtained by MIP, as shown in Figure 7. Compared with the blank group (FAA1.0) without zeolite (Figure 7a), under saturated steam conditions, the cumulative pore volume increased significantly due to the appearance of analcime-C. Moreover, due to the increase in the yield of analcime-C after changing conditions (Figure 7b–d), the smaller pores (10,000 nm) gradually transformed into larger pores (about 100,000 nm), and the pore size distribution became more extensive. In terms of mesopores and microporous structure, saturated steam-treated samples also showed similar advantages, as shown in Figure 8. Although the cumulative pore volume of sample AF1-2 (Figure 8d) is slightly lower than that of sample AF1-1 (Figure 8c), this may be due to the fact that more large pores are broken as the crystallization time increases, but its microporous structure is more abundant. The basic microstructural parameters such as BET surface area and DFT cumulative pore volume of FAA1.0, AF0.5-2, AF1-1, and AF1-2 are summarized in Figure 9. Surface area generally decreased with increasing pore size, so the higher microporous cumulative pore volume in sample AF1-2 provided a larger specific surface area. As shown in Figure 9, the samples with relatively wide pore size distribution showed higher surface area and pore volume, possibly due to the hierarchical connectivity of pores. Combined with the SEM images, it can be seen that various types of cracks and connected pores created interstitial pores in the range of micropores and mesopores. The increase in specific surface area and pore volume was due to the hierarchical connectivity of these interstitial pores. In conclusion, samples showing high micropores and mesopores volume were favorable for adsorption applications.

**Figure 10:**
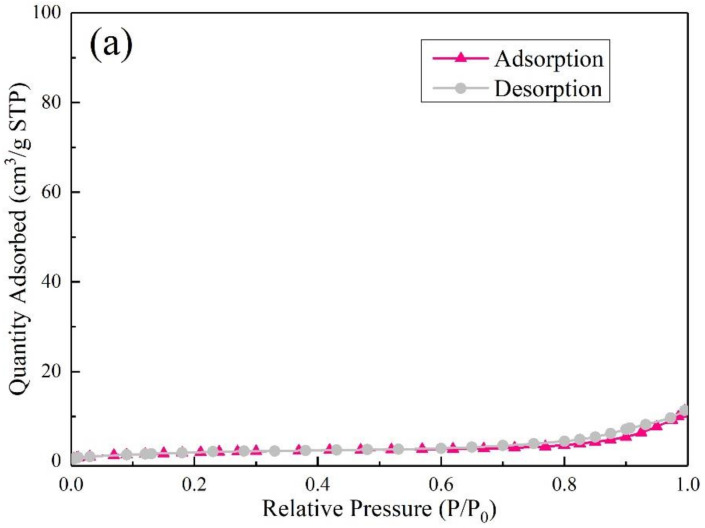

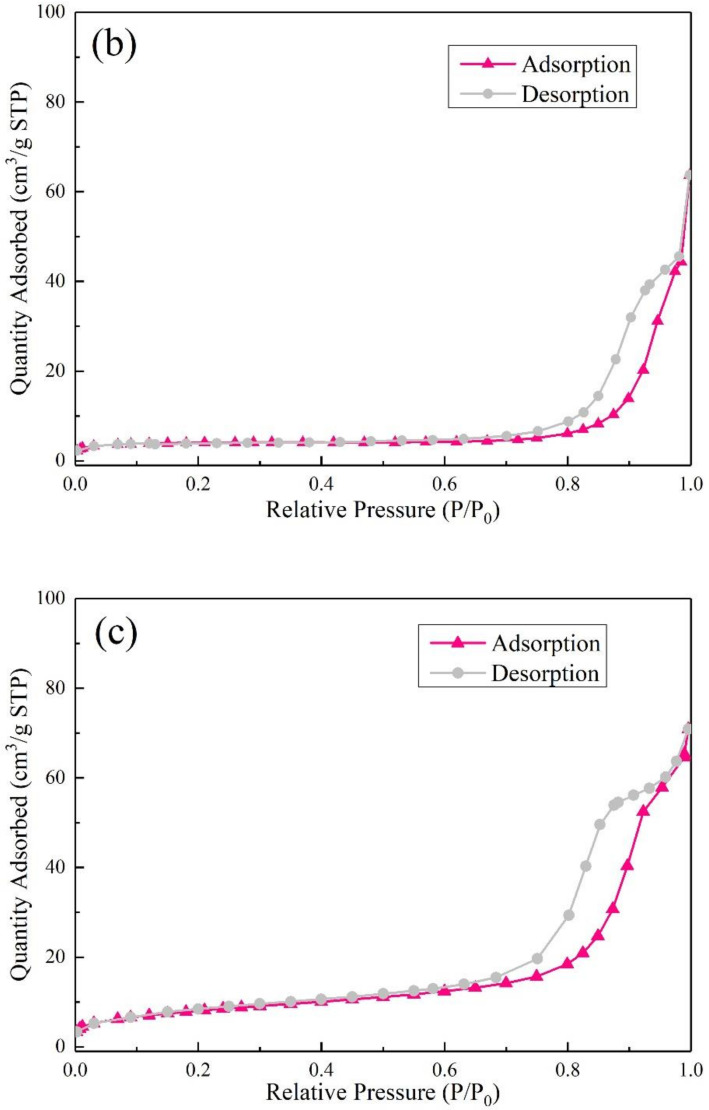

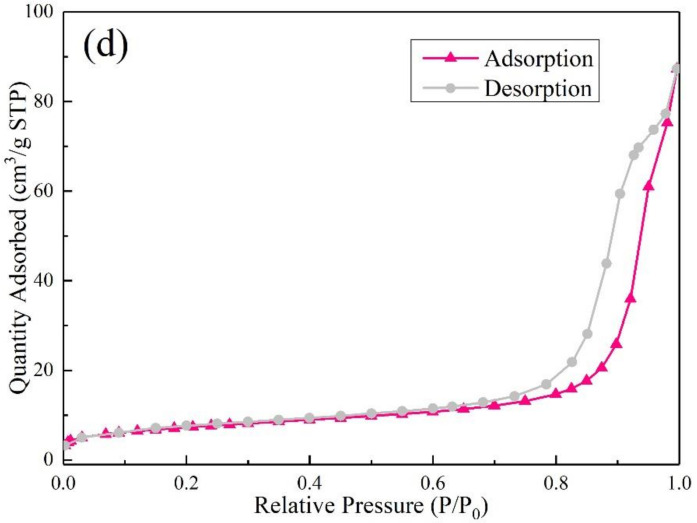
N_2_ adsorption and desorption isotherms at 77 K of (**a**) FAA1.0, (**b**) AF0.5-2, (**c**) AF1-1, and (**d**) AF1-2.

The N_2_ adsorption–desorption isotherms of above-mentioned four samples are plotted in Figure 10. According to IUPAC classification, these isotherms can be classified into six types (I to VI) with five types of hysteresis loops (H1 to H5) [44], All the samples exhibited type IV isotherms with H4 type hysteresis loops, which are the characteristic of many industrial mesoporous adsorbents [44].

In summary, the AFs had better microstructure characteristics in pore size distribution, specific surface area, and pore volume than the blank control. At the same time, to meet certain strength requirements, the higher the content of analcime-C, the better the pore characteristics of AFs.

### 3.4. Pb^2+^ Adsorption Tests

**Figure 11:**
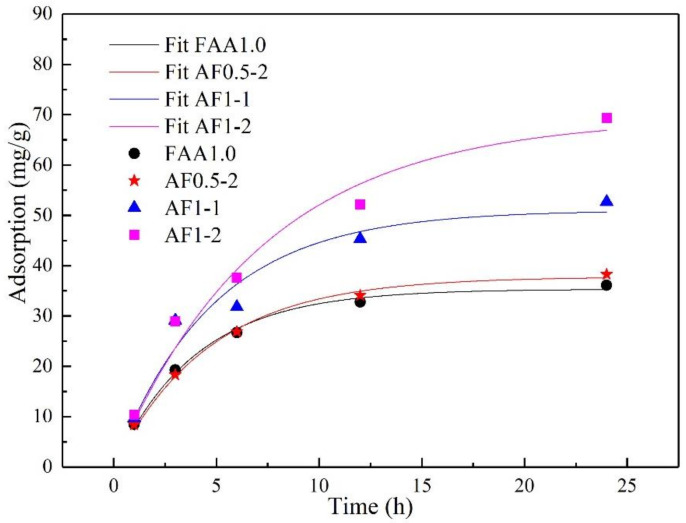
Pb^2+^ adsorption capacity of FAA1.0, AF0.5-2, AF1-1, and AF1-2.

As shown in Figure 11, All the samples showed relatively higher adsorption rate during first few hours of the adsorption experiment, and then started to slow down as the experiments continued, similar to many studies on the adsorption of lead by zeolite and AAM [35,36]. Sample AF1-2 showed the highest lead adsorption capacity of 69.3 mg/g at 24 h, followed by sample AF1-1. Samples FAA1.0 and AF0.5-2 only showed 36.1 mg/g and 38.3 mg/g adsorption capacity, respectively. It was evident that the analcime-C increased the adsorption potential of the AFs. However, it should be noted that in AFs, adsorption did not only occur simply by ion-exchange (mainly with Na^+^) or selective sieving through analcime-C [36]. In fact, N(C)-A-S-H gels and other amorphous phase which were major part of samples also contributed to adsorption, as reported in the studies on N(C)-A-S-H based adsorbents [30,43]. Higher N(C)-A-S-H gels and amorphous phase content possibly resulted in higher adsorption capacity of sample AF0.5-2 compared to sample FAA1.0. Moreover, considering that AFs were porous bulk-type solid adsorbents, hence, the macrostructure and microstructural characteristics (pore size, pore volume, and surface area) also affected the adsorption capacity. Similar findings have been reported in the literature [45,46], that the heavy metal ion loading increased with pore size, pore volume, and surface area. This behavior could be due to Pb^2+^ easily diffusing through the more abundant porous structure. According to the BET and the MIP analysis, we found that the sample AF1-2 had the most extensive pore distribution, the largest specific surface area, and the highest pore volume. This could be why the AF1-2 sample had the largest adsorption capacity.

In Table 4, comparison of some of the adsorbents for Pb^2+^ removal was shown. Although the AFs adsorption capacities were lower than the geopolymer solid powder, they were much higher than the adsorption capacities of geopolymer piece, self-supporting zeolite-type materials, and FAAs, according to previous reports. At the same time, AFs added advantage of light weight means they could be easily collected after use, unlike granular adsorbents.

## 4. Conclusions

We used a by-product of the lithium carbonate industry to synthesize an AAM-supported analcime-C foaming material which combined the excellent properties of AAM, zeolite, and foaming materials. At the same time, the conditions of the saturated steam were consistent with current industrial autoclaved aerated concrete blocks, which facilitates the industrialization of the material. Meanwhile this material would reduce environmental pollution, with an added advantage that these lightweight bulk-type AFs could be easily retrieved after use unlike granular adsorbents.

We can draw the following conclusions:

(1) From XRD results, it was confirmed that the analcime-C was found to be mainly in the zeolitic phase. Synthesis parameters, such as saturated steam temperature, pressure, and time, had large effects on compressive strength and the yield of analcime-C.
(2) MIP, BET, and SEM results illustrated that the AFs combined micro-porosity, meso-porosity, and macro-porosity. We obtained materials with pores ranging from the micro- to the macro range. The phase formation of analcime-C was partial, resulting in voids in the backbone, which enriched the porous structure of the materials.
(3) The Pb^2+^ adsorption test showed that the AFs materials had excellent ability in the removal of heavy metal ions (Pb^2+^), exhibiting 69.3 mg/g adsorption capacity. Its adsorption potential was higher than the FAAs and was also higher than the geopolymer pieces and self-supported zeolite materials reported in other studies.

## Figures and Tables

**Table 1:** Chemical compositions of MLS (wt%).

Oxide	SiO_2_	Al_2_O_3_	SO_3_	CaO	Fe_2_O_3_	K_2_O	others	LOI
MLS	59.5	24.9	7.68	5.42	1.68	0.484	0.336	1.03

LOI: Loss on ignition.

**Table 2:** The specific synthesis conditions of AFs.

Mark	MLS (g)	AA (g)	Modulus (n)	Pressure (MPa)	Temperature (°C)	Time (h)
AFn1.4	400	347	1.4	1	179.88	2
AFn1.2	400	311	1.2	1	179.88	2
AFn1.0 (AF1-2)	400	275	1	1	179.88	2
AF0.5-2	400	275	1.0	0.5	151.85	2
AF2-2	400	275	1.0	2	212.37	2
AF1-1	400	275	1.0	1	179.88	1
AF1-4	400	275	1.0	1	179.88	4

Note: Temperature, pressure, and time conditions of the saturated steam treatment.

**Table 3:** Rietveld quantitative analysis results of AFs (%).

Sample	Quartz	Lithium Aluminum Silicate/Spodumene	Sodium Sulfate	Calcium Sulfate	Amorphous	Analcime-C	Rwp * (%)
AFn1.4	4.28	2.41	-	0.01	84.06	9.24	7.24
AFn1.2	6.08	3.03	-	0.01	83.17	7.71	6.00
AFn1.0 (AF1-2)	2.64	-	-	-	58.57	38.79	8.46
AF0.5-2	3.15	3.91	0.04	0.07	92.83	-	8.03
AF2-2	4.70	-	-	-	46.41	48.89	8.51
AF1-1	3.03	2.04	-	0.01	80.98	13.94	8.42
AF1-4	4.16	-	-	-	50.63	45.21	8.13

*: Rwp is a refinement numerical criterion. It is generally considered that Rwp is less than 10%, and the refinement result is reliable.

**Table 4:** Pb^2+^ adsorption capacity onto various adsorbents.

Adsorbent	Q (mg/g)	pH	Time (h)	Temperature (°C)	Reference
Geopolymer piece	6.3	5	24	25	[29]
Self-supported zeolites	26.1	5	24	25	[28]
FAAs	36.1	5	24	25	[32]
AFs	69.3	5	24	25	This study
Fly ash-based geopolymer powder	91	5	24	25	[30]

## Data Availability

Data sharing applicable.
